# Mechanical Stretch Induced Osteogenesis on Human Annulus Fibrosus Cells through Upregulation of BMP-2/6 Heterodimer and Activation of P38 and SMAD1/5/8 Signaling Pathways

**DOI:** 10.3390/cells11162600

**Published:** 2022-08-20

**Authors:** Cheng-Nan Chen, Hsin-I Chang, Chia-Kung Yen, Wen-Lung Liu, Kuo-Yuan Huang

**Affiliations:** 1 Department of Biochemical Science and Technology, National Chiayi University, Chiayi City 60004, Taiwan; 2 Department of Food Science, National Chiayi University, Chiayi City 60004, Taiwan; 3 Department of Orthopedics, National Cheng Kung University Hospital, College of Medicine, National Cheng Kung University, Tainan 70403, Taiwan

**Keywords:** degenerative disc disease, tensile stress, bone morphogenetic protein-2/6 (BMP-2/6) heterodimers, osteogenesis, p38 MAP kinase pathway, BMP-SMAD1/5/8 signaling

## Abstract

Degenerative disc disease (DDD) is an important cause of low back pain. Repetitive tensile stress from the daily motion of the spine predisposes it to injury of the annulus fibrosus (AF) which causes IVD degeneration. This study aims to determine the causal relationship between mechanical stretch and osteogenesis in the AF cells of IVD as affected by bone morphogenic proteins (BMPs), specifically BMP-2/6 heterodimers. Our results found that 15% tensile stress (high cyclic stretching, HCS) may induce the expression of osteogenesis-related markers (Runx2, osterix) by upregulating BMP-2/6 heterodimeric ligands and their receptors on the human AF cell line. HCS also induced transient phosphorylation of p38 mitogen-activated protein (MAP) kinase and SMAD1/5/8. Neutralizing antibodies to the BMP-2/6 receptor (ALK3) blocked the expression of Runx2 and osterix, as well as the phosphorylation of p38 and SMAD1/5/8. In addition, treatment with a p38 MAPK inhibitor (SB203580) or siRNA to neutralize the effects of SMAD1/5/8 suppressed tensile stress-induced Runx2 and osterix expression. Mechanical stretching induces activation of p38 MAP kinase and SMAD1/5/8 signaling pathways, followed by the upregulation of BMP-2/6 heterodimer expression, thereby stimulating osteogenic Runx2 and osterix expression on AF cells. HCS may accelerate the progression of IVD degeneration by promoting an osteogenic response.

## 1. Introduction

Degenerative disc disease (DDD) is a significant cause of low back pain [1,2,3]. The prevalence of DDD is roughly proportional to age, for example, 40% of people in their 40 s have DDD, and this rate increases to 80% in people aged 80 years or older [4]. DDD seriously affects people’s quality of life and ability to work, causing a huge medical burden; the disease has become a global socioeconomic problem [5]. The pathogenesis of DDD is a complex disease, including excessive mechanical loading, trauma, inflammation, genetic predisposition, aging, obesity, limited nutrition, acidic pH of disc environment, etc. [6,7,8,9,10,11,12,13].

Physical damage to the annulus fibrosus (AF) of the intervertebral disc (IVD) is an important cause of spinal degeneration [14]. As a major component of IVD, AF acts as a useful buffer against mechanical loads caused by spinal movement [15]. However, excessive mechanical stretching changes the number and phenotype of AF cells, thereby reducing the elasticity of the AF, leading to further IVD damage and degeneration [12,16,17]. Furthermore, AF cells possess the properties of progenitor cells and can differentiate into chondrocytes and osteoblasts in vitro and in vivo under chondrogenic and osteogenetic medium stimulation. In the injured rat intervertebral discs, bone formation and hypertrophic chondrocytes were observed in AF tissue [18]. Therefore, it is important for us to investigate the signaling pathway of osteogenesis on AF cells in order to elucidate the pathogenesis of DDD.

Excessive mechanical stimulation reduces substrates produced by AF cells, leading to IVD dehydration [19], and a higher 12% cyclic tensile stress has been reported to inhibit AF cell proliferation and increase the expression levels of inflammatory genes [20]. Abnormal mechanical loading also affects the metabolism of AF cells, resulting in structural damage leading to IVD degeneration [21,22]. Thus, mechanical force may play a critical role in the homeostasis of AF cellular structure and function [12,23,24]. Excessive mechanical stretching has also been shown to enhance the inflammatory responses in IVDs [20], thereby inducing the expression of matrix-degrading enzymes and inhibiting extracellular matrix synthesis [25,26], ultimately accelerating IVD degeneration [12,23,24]. It is well known that stem or progenitor cells reside inside IVDs and their endplates [27,28]. Interestingly, mechanical forces such as cyclic compression and shear stress are able to stimulate bone morphogenetic protein-2 (BMP-2) and osteopontin (OPN) expressions in bone marrow-derived human mesenchymal stem cells (MSCs) [29,30]. Furthermore, our group has found enhanced corresponding osteogenic and degenerative responses, such as subchondral sclerosis, endplate hypertrophy, or osteophyte formation after BMP-2 treatment in a rabbit in vivo model of intervertebral disc injury [31], strongly suggesting that BMPs play a central role in the pathogenesis of DDD [31,32].

BMPs are members of the transforming growth factor-beta (TGF-beta) superfamily with osteoinductive potential to induce the differentiation of stem cells into osteoblasts and promote bone formation [33,34]. BMPs are expressed in IVD cells [35,36] and can be used to promote spinal fusion [32,37,38] and play an important role in fracture healing [39]. The expressions of BMP-2 and its type I and II receptors have been observed in fibrous cells in the IVD of mice [40]. BMP-2/4 and their type I and II receptors were vigorously expressed in fibrous cartilaginous cells within AF in senescence-accelerated mice aged 50 weeks and thus seemed to be related to degeneration of IVD [41]. We also found that BMP-2 synergized with the inflammatory cytokines IL-1β and IL-20 to regulate the healing process after human IVD herniation [42,43,44]. So far, many studies have used heterodimeric ligands comprising two BMP genes to elucidate the broad biological roles of BMP heterodimers, and co-expression of different BMP ligands can lead to heterodimeric ligand formation. Heterodimeric BMPs are more active than their homodimeric counterparts in the bone formation processes. In addition, heterodimers such as BMP-2/6 are more efficient than BMP-2 or BMP-6 homodimers in human embryonic stem cell differentiation [45]. However, the roles of BMP-2/6 heterodimers have not been well studied in the pathogenesis of IVD degeneration. Therefore, the aim of this study is to determine the role of the BMP-2/6 heterodimer in IVD and the causal relationship between mechanical stretching and osteogenic response in IVD as well as signaling pathways in IVD degeneration.

## 2. Materials and Methods

### 2.1. Patients

This study enrolled ten patients (mean age 41.2 years, range 26–55 years) who were diagnosed with DDD of lower lumbar or lumbosacral spine, with Pfirrmann disc degenerative grade 4 or 5 in our department, and all of them underwent posterior laminectomy for decompression, posterior internal fixation, posterior lateral fusion, and vertebral interbody fusion. All patients had low back pain, claudication, or sciatica. We collected twelve clinical samples of AF and ligamentum flavum (LF) tissues from patients. All patients received supine X-rays and lateral lumbosacral X-rays with dynamic flexion and extension. Magnetic resonance imaging studies are used to confirm the diagnosis of DDD. All patients tried conservative treatment but failed, and all agreed to undergo surgical intervention. Our institutional review board approved this study and informed consent was obtained from all participants. Written informed consent was obtained. The study was conducted according to approved guidelines.

### 2.2. Reagents 

Culture materials and fetal bovine serum (FBS) were purchased from Gibco (Grand Island, NY, USA). MAPK inhibitors including PD98059 (inhibitor for ERK), SP600125 (inhibitor for JNK), SB203580 (inhibitor for p38), and LDN193189 (inhibitor for ALK3) were purchased from Calbiochem (La Jolla, CA, USA). Antibodies against p38, phospho-p38, SMAD1/5/8, phospho-SMAD1/5/8, osterix, and OPN were purchased from Santa Cruz Biotechnology (Santa Cruz, CA, USA). Rabbit polyclonal antibody against Runx2 was purchased from Cell Signaling Technology (Beverly, MA, USA). The catalog numbers and dilutions for antibodies used in Western blot analysis were provided in Table S1. Neutralizing mABs against BMP-2, BMP-6 were purchased from R&D Systems (Minneapolis, MN, USA). Runx2, SMAD1 siRNA vectors, and a control siRNA construct (containing random DNA sequences) were purchased from Invitrogen (Carlsbad, CA, USA). All other chemicals of reagent grade were obtained from Sigma (St. Louis, MO, USA).

### 2.3. Culture of Cell Line of AF Cells

Human AF cells were purchased from ScienCell Research Laboratories (Carlsbad, CA, USA) twice during the experiment. The cells were incubated in a complete growth medium supplemented with 10% FBS. Cells in passages 1–4 were subcultured and preserved for stock. Most of the experiments were performed with cells in passages 5–6. After reaching 80% confluency, the cells were trypsinized and seeded onto stretch chambers.

### 2.4. Cyclic Stretching

Commercial silicone stretching chambers (4 cm^2^, STB-CH-10, STREX) were passively coated with 50 µg/mL fibronectin (FC010, Merck Millipore, MA, USA) at 37 °C for 24 h. The human AF cells at passages 5–6 were seeded in the silicone chambers (1 × 10^5^ cells per chamber in 5 mL growth medium) and cultured at 37 °C, 5% CO_2_ for 3 days, unless otherwise stated. The cells were cultured in a serum-free medium for 12 h before being applied to stretching apparatus. The AF cells cultured in chambers were mounted on a commercial stretching bioreactor (STB-140-10, STREX) and subjected to 5% (light cyclic stretch, LCS) or 15% (high cyclic stretch, HCS) cyclic sinusoidal uniaxial strain at60 cycle/min at 37 °C and 5% CO_2_. Cells were stretched for designated time courses and time-matched control chambers were kept in static conditions without stretching. Immediately after the mechanical loading, the cells were lysed for gene and protein expression analysis.

### 2.5. Real-Time Quantitative PCR

For gene expression analysis, AF cells (1 × 10^5^ cells/chamber) are cultured on a stretched matrix with an area of 4 cm^2^ for 72 h and then cyclic stretched for the time as indicated. Total RNA was extracted using Trizol reagent (Thermo Fisher Scientific, Waltham, MA, USA), and reverse transcription reactions were performed as described previously [46]. Gene expression was analyzed by quantitative real-time PCR on a 7900HT real-time PCR system (Applied Biosystems, Foster City, CA, USA) using the SYBR Green PCR Master Mix (Applied Biosystems). Real-time PCR reactions were performed in triplicate and relative changes in mRNA expression were quantified using the 2^−ΔΔCt^ method normalized to GAPDH [46]. The specific sequences of BMP-2 primer (plus-CGC AGC TTC CAC CAT GAA GAA and minus-CCT GAA GCT CTG CTG AGG TGA TA), BMP4 primer (plus-AGG AGC TTC CAC CAC GAA GAAC and minus-TGGA AGC CCC TTT CCC AAT CAG), BMP-6 primer (plus-GTG AAC CTG GTG GAG TAC GACAA and minus-AGG TCA GAG TCT CTG TGC TGATG), BMP7 primer (plus-ACC AGA GGC AGG CCT GTA AGA and minus-CTC ACA GTT AGT AGG CGG CGT AG), OPN primer (plus-GGA CAG CCAG GAC TCC ATTG and minus-TGT GGG GAC AAC TGG AGT GAA), Runx2 primer (plus-GCC TTC AAG GTG GTA GCC C and minus-AAG GTG AAA CTC TTG CCT CGT C), osterix primer (plus-CCT GCG ACT GCC CTA ATT and minus-GCG AAG CCT TGC CAT ACA), and GAPDH primer (plus-AGG TGA AGG TCG GAG TCA AC and minus-CCA TGT AGT TGA GGT CAA TG AAGG) were used in the real-time PCR. The expression level of GAPDH was used as the internal control.

### 2.6. Western Blot Analysis

For Western blot analysis, AF cells (1 × 10^5^ cells/chamber) are cultured on a stretched matrix with an area of 4 cm^2^ for 72 h and then cyclic stretched for the time as indicated. For each Western blot analysis, cells were collected and mixed from two stretching experiments. Cells were lysed with a lysis buffer (1% NP-40, 0.5% sodium deoxycholate, 0.1% SDS, and a protease inhibitor mixture). After protein quantification, the cell lysate (50 μg of protein) was separated by SDS-polyacrylamide gel electrophoresis (PAGE) (12% running, 4% stacking) and analyzed using the target protein antibodies and the Western-Light chemiluminescent detection system (Bio-Rad, Hercules, CA, USA), as previously described [46].

### 2.7. siRNA Transfection

Cells were transfected with the designated construct using an RNAiMAX transfection kit (Invitrogen). Target gene (Runx2-, SMAD-, ALK3-siRNA) transfections caused at least an 80% reduction in the respective protein expression levels compared with the siRNA control vector (data not shown). The following RNA primers sequence (siRNA) were used: Runx2: 5′-UAGUG GCAGAAUGGAUGAAUCUGUU-3′; Smad1: 5′-GCAACCGAGUAACUGUGUCACCAUU-3′; ALK3: 5′-GGAUACCUUGCCUUUUUUA-3′. The target mRNA levels were measured after 48 h transfection.

### 2.8. Statistical Analysis

The results are expressed as mean ± standard deviation (SD) in this study. Analysis of variance (ANOVA) followed by Scheffe’s test for multiple comparisons was used to evaluate the statistical significance. *p* values less than 0.05 were considered significant. 

## 3. Results

### 3.1. HCS Upregulates Osteogenic Gene Expression in Human AF Cells

**Figure 1:**
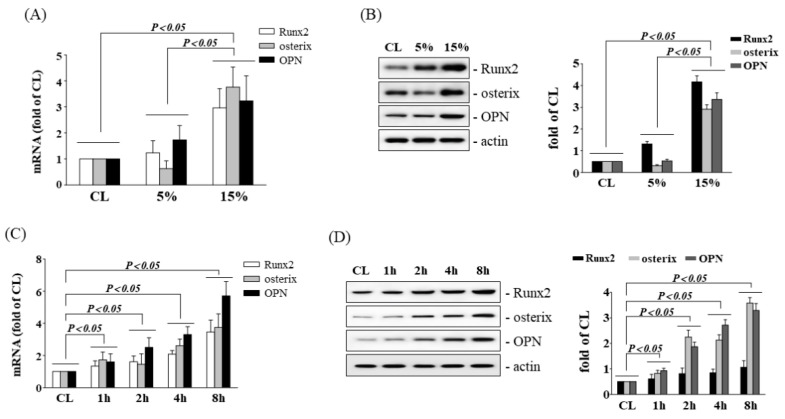
HCS upregulates osteogenic gene expression in human AF cells. (**A**,**B**) AF cells were kept in static condition as controls (CL) or subjected to LCS (5%) or HCS (15%) for 8 h, and the gene expressions of Runx2, osterix, and OPN (**A**) were determined by real-time PCR analysis. The protein expressions were determined by Western blotting (**B**). (**C**,**D**) AF cells were subjected to HCS for the indicated times, and the gene expression of Runx2, osterix, and OPN. (**A**,**C**) were determined by real-time PCR analysis. (**B**,**D**) The protein expressions were examined by Western blotting. The results are mean ± standard deviation (SD) and are representative of three independent experiments (n = 3). Data statistics were completed with ANOVA followed by Scheffe’s test. Results were considered significant for *p* < 0.05.

To test whether cyclic stretch could initiate gene expression associated with IVD osteogenesis, human AF cells were cultured and subjected to cyclic stretch protocols, in each of which LCS (5%) or HCS (15%) was applied with different resting periods as shown in Figure 1. We examined whether HCS treatment was capable of inducing Runx2, osterix, and OPN expressions in AF cells. The AF cells were either maintained as controls or were subjected to LCS or HCS for different time courses. The cells stimulated by HCS for 4 h significantly induced Runx2, osterix, OPN mRNA (Figure 1A), and protein (Figure 1B) expressions in AF cells in comparison with the control and LCS-treated cells. In addition, the time courses determined for the Runx2, osterix, OPN mRNA (Figure 1C), and protein (Figure 1D) levels revealed an increase within 1 h and persisted for 8 h in AF cells of HCS treatment.

### 3.2. Involvement of Runx2 in HCS-Induced Osteogenesis

**Figure 2:**
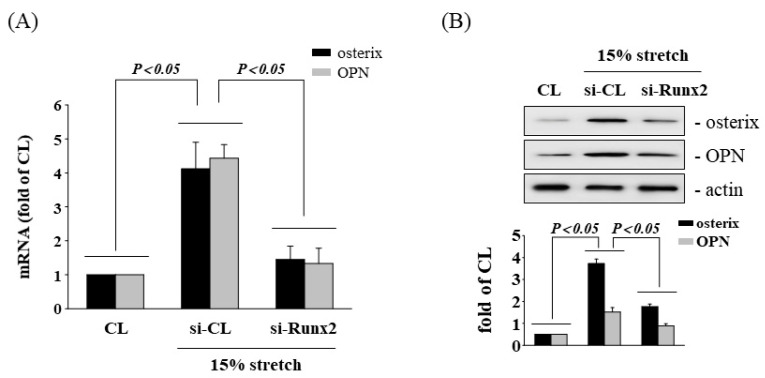
Involvement of Runx2 in tensile stretch-induced osteogenesis. AF cells were kept in static condition as CL or subjected to HCS for 8 h. Before being kept as CL or subjected to HCS, AF cells were transfected with control siRNA (si-CL), or a specific siRNA of si-Runx2. (**A**) All bar graphs represent multiple increases in mRNA expression of CL AF cells normalized to 18S rRNA. (**B**) The protein expression was examined by Western blot analyses. The results are mean ± SD and are representative of three independent experiments (n = 3). Data statistics were completed with ANOVA followed by Scheffe’s test. Results were considered significant for *p* < 0.05.

Runx2 is a key transcription factor for osteogenic gene expression and hence we investigated whether Runx2 could regulate the HCS effect on osterix and OPN expression in AF cells. The AF cells were maintained either as controls or were subjected to HCS for 4 h, after which we examined the osterix and OPN expression. Pretreating cells with Runx2-specific siRNA significantly inhibited HCS-induced osterix and OPN mRNA (Figure 2A) and protein (Figure 2B) expression in the AF cells.

### 3.3. BMP-2/6 Heterodimer Mediates Stretch-Induced Osteogenic Gene Expression

**Figure 3:**
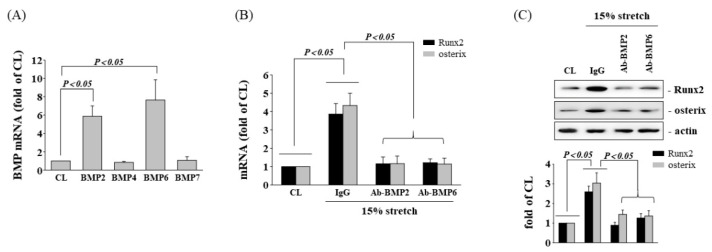
BMP-2/6 heterodimer mediates tensile stretch-induced osteogenic gene expression. (**A**) The AF cells were kept in static condition as the control or were subjected to HCS for 4 h, after which the BMP-2/4/6/7 mRNA expression in AF cells was determined by real-time PCR analysis. (**B**,**C**) AF cells were maintained as CL or subjected to 15% HCS for 8 h. Before being kept as CL or exposed to CS, AF cells were pretreated with isotype-matched IgG or neutralizing antibodies against BMP-2 (Ab-BMP-2) and BMP-6 (Ab-BMP-6). The expression levels of Runx2 and osterix mRNA (**B**) were measured by real-time PCR analysis. The protein expressions were examined by Western blotting (**C**). The results are mean ± SD and are representative of three independent experiments (n = 3). Data statistics were completed with ANOVA followed by Scheffe’s test. The results were considered significant for *p* < 0.05.

**Figure 4:**
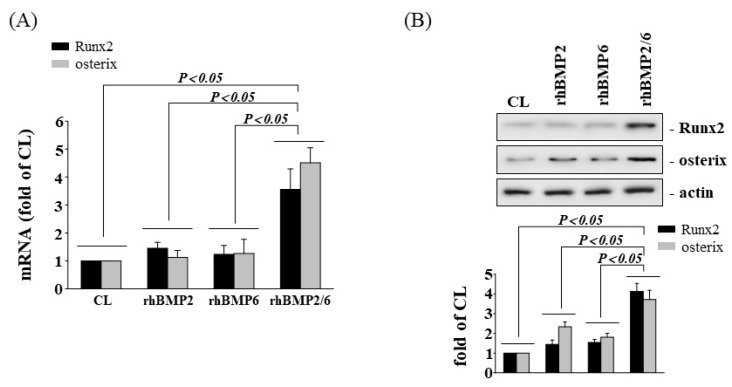
Recombinant human (rh)BMP-2/6 heterodimer upregulates osteogenic gene expression. AF cells were kept as controls (CL) or treated with recombinant proteins of BMP-2, BMP-6, or BMP-2/6 heterodimer for 8 h, and the gene expressions of Runx2 and osterix (**A**) were measured by real-time PCR analysis. The Runx2 and osterix protein expressions were examined by Western blotting (**B**). The results are mean ± SD and are representative of three independent experiments (n = 3). Data statistics were completed with ANOVA followed by Scheffe’s test. The results were considered significant for *p* < 0.05.

BMPs are major regulators of osteogenic differentiation. Therefore, we set out to determine whether HCS treatment-initiated osteogenic gene expression would arise through upregulating the BMPs in human AF cells. Cells were either kept in static condition as the control or were subjected to HCS, after which we examined the gene expressions of BMPs in the AF cells. The cells subjected to HCS significantly increased the mRNA expression of BMP-2 and BMP-6 in AF cells in comparison with the control (Figure 3A). In addition, the cells pretreated with neutralizing antibodies against BMP-2 (Ab-BMP-2) and BMP-6 (Ab-BMP-6) significantly suppressed the up-regulatory effects of HCS on the gene (Figure 3B) and protein (Figure 3C) expressions of Runx2, osterix, and OPN in AF cells. In the recombinant protein experiments, neither BMP-2 nor BMP-6 could upregulate the gene and protein expressions of Runx2 and osterix, while BMP-2/6 induced significant expression of Runx2 and osterix at the same concentration (Figure 4A,B). This result speculates that BMP-2/6 has been effective in inducing Runx2 and osterix expression when the concentration of BMP-2 or BMP-6 is not enough to induce gene and protein expressions.

### 3.4. HCS-Induced Runx2 Expression Is p38 MAPK Dependent

**Figure 5:**
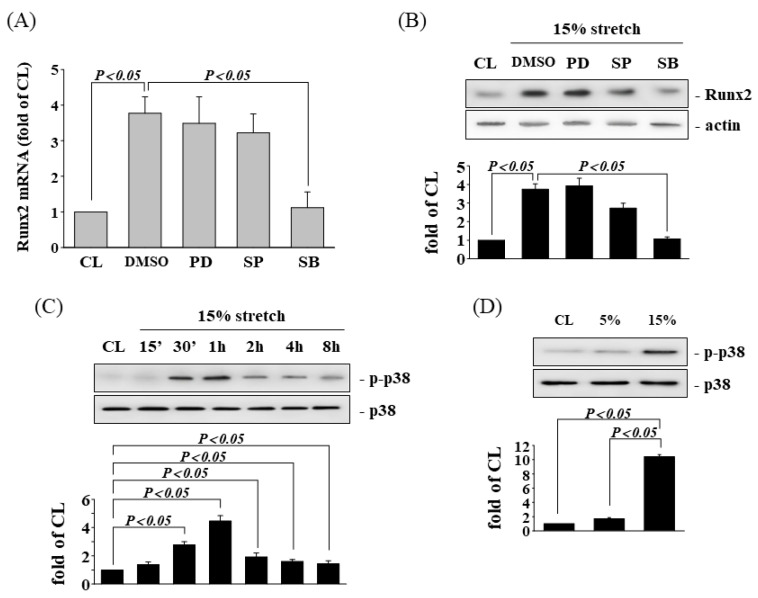
HCS-induced Runx2 expression is p38 MAPK dependent. AF cells were maintained in static conditions as CL or subjected to HCS for 8 h. Before being kept as CL or subjected to CS, AF cells were pretreated with DMSO, or specific kinase inhibitors for ERK1/2 (PD98059, 30 μM), JNK (SP600125, 20 μM), or p38 (SB203580, 10 μM) for 1 h and then subjected to HCS for 8 h. The mRNA (**A**) and protein (**B**) expression of Runx2 were determined by real-time PCR and Western blotting, respectively. (**C**) Control or HCS-stimulated AF cells were maintained for the times indicated, and the phosphorylation of p38 in these cells was determined using Western blotting. (**D**) The phosphorylation of p38 in AF cells after 1 h of LCS (5%) or HCS (15%) stimulation was examined using Western blotting. The results are mean ± SD and are representative of three independent experiments (n = 3). Data statistics were completed with ANOVA followed by Scheffe’s test. The results were considered significant for *p* < 0.05.

The MAPK pathway has been shown to regulate a number of cellular processes, including osteogenic gene expressions. To investigate the involvement of MAPKs in the modulation of Runx2 expression by HCS induction, human AF cells were treated with specific inhibitors for MAPKs (PD98059 for ERK, SP600125 for JNK, SB203580 for p38) for 1 h before and during HCS stimulation. SB203580 was found to significantly inhibit the HCS-induced gene (Figure 5A) and protein (Figure 5B) expression of Runx2. The phosphorylation of p38 in AF cells increased rapidly after HCS induction, reaching maximal levels at 1–2 h (Figure 5C). Furthermore, the cells stimulated by HCS for 1 h significantly induced p38 phosphorylation in AF cells in comparison with the control and LCS-treated cells.

### 3.5. SAMD1/5/8 Is Involved in Tensile Stretch-Induced Runx2 Expression

**Figure 6:**
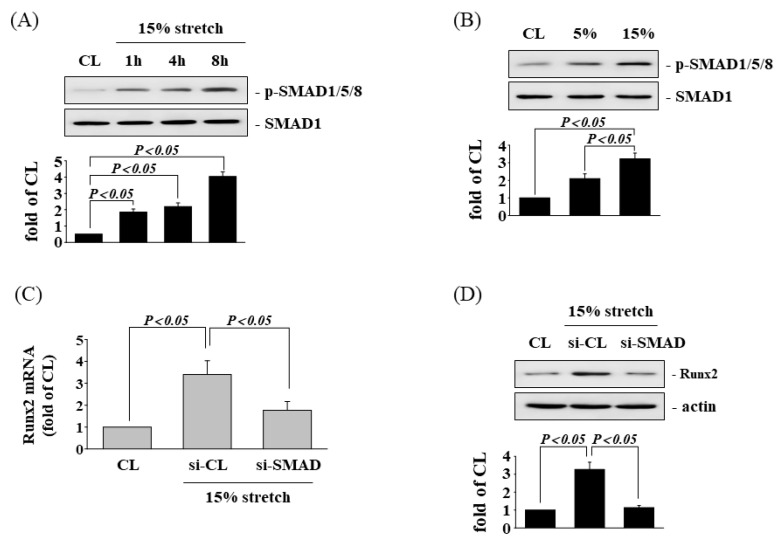
SAMD1/5/8 is involved in HCS-induced Runx2 expression. (**A**) Control or HCS-stimulated AF cells were maintained for the times indicated, and the phosphorylation of SMAD1/5/8 in these cells was determined using Western blotting. (**B**) The phosphorylation of SMAD1/5/8 in AF cells after 1 h of LCS (5%) or HCS (15%) stimulation was determined using a Western blot analysis. The results shown are representative of three independent experiments that gave similar results. (**C**,**D**) AF cells were kept as CL or subjected to 15% HCS for 8 h. Before being kept as CL or subjected to CS, AF cells were transfected with control siRNA (si-CL), or a specific siRNA of si-SMAD, and then exposed to HCS for 8 h. The mRNA (**C**) and protein (**D**) expression of Runx2 were examined by real-time PCR and Western blotting, respectively. The results are mean ± SD and are representative of three independent experiments (n = 3). Data statistics were completed with ANOVA followed by Scheffe’s test. The results were considered significant for *p* < 0.05.

The SMAD proteins that are required for the transmission of the BMP signals to the nucleus have been shown to induce osteogenic gene expression. We investigated whether SMAD1/5/8 could regulate the HCS-induced Runx2 expression in human AF cells. The AF cells were maintained either as controls or were exposed to HCS for 1, 4, and 8 h, after which we determined the SMAD1/5/8 phosphorylation. HCS-treatment of the AF cells significantly induced SMAD1/5/8 phosphorylation within 1 h and persisted for 8 h in comparison with the control (Figure 6A). The human AF cells stimulated by HCS for 4 h significantly induced SMAD1/5/8 phosphorylation when compared to the control and LCS-treated cells (Figure 6B). Pretreating cells with SMAD1-specific siRNA significantly inhibited HCS-treatment-induced Runx2 mRNA (Figure 6C) and protein (Figure 6D) expression.

### 3.6. Inhibition of ALK3 Receptor Decrease Tensile Stretch-Induced Osteogenic Gene Expression

**Figure 7:**
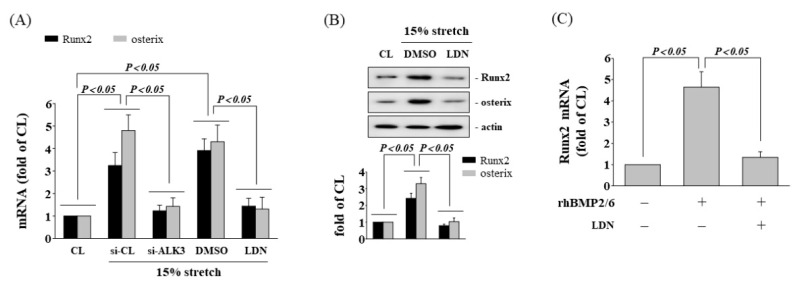
Inhibition of ALK3 receptor decreases HCS-induced osteogenic gene expression. AF cells were maintained in static conditions as CL or subjected to 15% HCS for 8 h. Before being kept as CL or subjected to CS, AF cells were transfected with control siRNA (si-CL), or a specific siRNA of si-SMAD, or were pretreated with DMSO, or specific kinase inhibitors for ALK3 (LDN) for 1 h, and then subjected to 15% HCS for 8 h. The gene (**A**) and protein (**B**) expression of Runx2 were examined by real-time PCR and Western blotting, respectively. The results are shown as mean ± SD. (**C**) AF cells were kept as CL or stimulated with rhBMP-2/6. Before being kept as CL or stimulated with rhBMP-2/6, AF cells were pretreated with DMSO, or specific kinase inhibitors for ALK3 (LDN) for 1 h, and then stimulated with rhBMP-2/6 for 8 h. The mRNA expression of Runx2 was measured by real-time PCR. The results are mean ± SD and are representative of three independent experiments (n = 3). Data statistics were completed with ANOVA followed by Scheffe’s test. The results were considered significant for *p* < 0.05.

It has been reported that BMP-2/6 heterodimer can bind and activate ALK3 receptors, and further affect host cell gene expression. To assess the role of ALK3 in osteogenic phenotypes of HCS-stimulated AF cells, we evaluated the effects of ALK3-siRNA and inhibitor (LDN) on HCS-induced Runx2 and osterix expression in human AF cells. The HCS-induced mRNA expression of Runx2 and osterix were significantly reduced by ALK3-siRNA or LDN treatment (Figure 7A). In addition, the HCS-induced protein expression of Runx2 and osterix were also suppressed in AF cells pretreated with LDN (Figure 7B). In addition, the Runx2 mRNA expression stimulated by rhBMP-2/6 heterodimer in AF cells was significantly inhibited in cells pretreated with LDN (Figure 7C).

### 3.7. Osteogenic Gene Expression in Patients with IVD Injury

**Figure 8:**
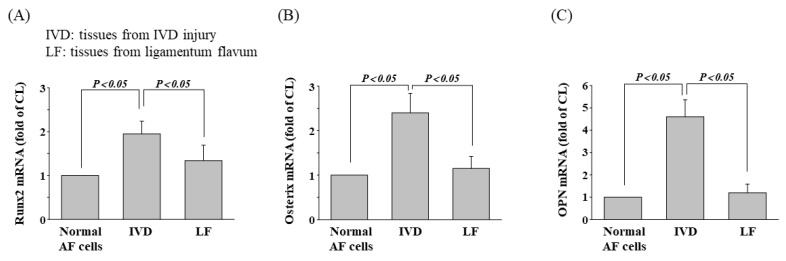
Osteogenic gene expression in patients with IVD injury. The mRNA expression levels of (**A**) Runx2, (**B**) osterix, and (**C**) OPN were measured by real-time PCR. IVD and LF tissue samples were collected from patients with DDD (IVD, N:12; LF, N:12). All experiments were performed three times. Data statistics were completed with ANOVA followed by Scheffe’s test. The results were considered significant for *p* < 0.05.

Figure 8 presents osteogenic gene expressions in tissues removed surgically from patients with IVD injury. Both degenerative IVD and LF tissues displayed osteogenic phenotypes, which was confirmed by an increase in the mRNA expression for Runx2 (Figure 8A), osterix (Figure 8B), and OPN (Figure 8C). The expression level of osteogenic genes was higher in tissues from AF than ones from LF.

## 4. Discussion

**Figure 9:**
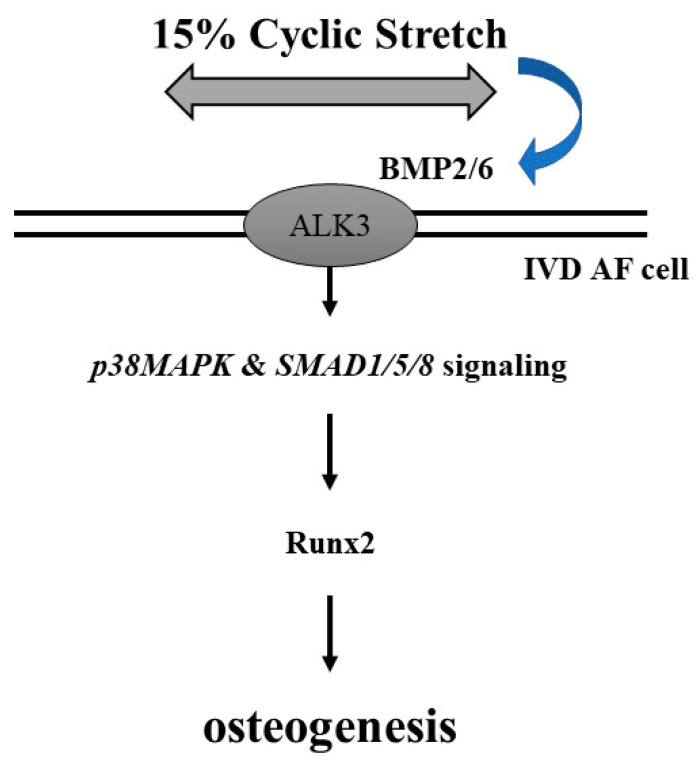
Schematic representation of a possible mechanism affecting 15% cyclic stretch-induced BMP-2/6 expression and consequent osteogenesis regulations in human AF cells.

In the present study, we investigated the expression levels of osteogenic genes such as Runx2, osterix, and OPN in AF cells that were subjected to LCS (5%) or HCS (15%) and found that HCS promoted the gene and protein expressions of Runx2, osterix, and OPN but not with LCS. Moreover, treatment of si-Runx2 blocked the response to the mechanical strength. The mRNA and protein expression of Runx2 and osterix in AF cells can be neutralized when pretreated with Ab-BMP-2 or Ab-BMP-6 and then stimulated with HCS. This suggests that both BMP-2 and BMP-6 are attributable to AF cell osteogenesis after HCS stimulation. In addition, AF cells treated with rhBMP-2/6 heterodimer significantly upregulated mRNA and protein expressions of Runx2 and osterix when the concentration of BMP-2 or BMP-6 is not enough to induce gene and protein expressions. Therefore, HCS contributes to the osteogenesis of AF cells through upregulation of the BMP-2/6 heterodimer. In addition, the osteogenic gene expressions, such as Runx2, stimulated by rhBMP-2/6 heterodimer in AF cells were modulated through p38 phosphorylation and SMAD1/5/8 activation. The proposed molecular mechanism for the impact of cyclic stretch-induced BMP-2/6 expression and consequent osteogenesis regulations on AF cells is illustrated in Figure 9.

NP, AF, and endplate in IVD are responsible for relieving mechanical stress. Each of these areas is functionally different, but all three tissues are critical to maintaining the structural integrity of the spine [19]. In the NP center, the main mechanical load is hydrostatic pressure because of the higher water content; whereas, AF cells are constantly exposed to cyclic tensile stress [19]. Focal biochemical and mechanical alterations decrease the ability of IVD to furnish its matrix components and ultimately diminish the efficiency of the IVD function. Feng et al. and others have reported that human AF cells could differentiate into multiple cell lineages under different stimulation, including adipocytes, chondrocytes, and osteoblasts [47]. Jin et al. also demonstrated that an osteogenic medium could induce osteogenic transformation of rabbit AF cells by increased mineralization and expressions of Runx2, BMP-2, and osteopontin genes [18]. On the other hand, Stich et al. used genome-wide microarray to evaluate the differences between human lumbar AF tissues from mild and severe degenerated IVDs and the results exhibited that Runx2 and other osteogenic-related genes were highly expressed in native AF tissues of severe degenerated IVD [48]. Similarly, Yeh et al. confirmed that human AF cells of degenerated IVDs exhibited a higher tendency to osteogenic differentiation over native AF cells by an increase in the expression of mRNAs for BMP-2, Runx2, ALP, and osteocalcin and calcium deposition over native AF cells [49]. Therefore, any alternation of the cellular environment may change IVD cell phenotypes and promote IVD degeneration.

Our study investigated the expression levels of osteogenic genes such as Runx2, osterix, and OPN in AF cells that were subjected to LCS (5%) or HCS (15%) and found that HCS promoted the gene and protein expressions of Runx2, osterix, and OPN but not with LCS. In the study of Rannou et al., mechanical loading with a 15% cyclic stretch significantly promoted the apoptosis of mouse AF cells, and that was reflected as the physiological limits of stress in IVD [50]. Therefore, we choose a 15% cyclic stretch as the high mechanical stress in this study. In addition, AF cells under HCS conditions could be transformed to the osteoblast-like phenotype, suggesting that exposure to high mechanical stress can drive AF cells to differentiate down the osteogenic lineage. Then, we assessed human AF cells cultured on stretch devices to investigate the effect of HCS on the signaling pathway leading to osteogenic Runx2 and osterix gene expression. Interestingly, AF cells stimulated with HCS were shown to increase the gene expressions of BMP-2 and BMP-6 but not BMP-4 and BMP-7. In addition, BMP-2 or BMP-6-specific siRNA significantly blocked the upregulation of Runx2 and osterix. On the other hand, neither rhBMP-2 nor rhBMP-6 could promote the gene and protein expression of Runx2 and osterix, while rhBMP-2/6 induced significant expression of Runx2 and osterix at the same concentration (Figure 4). This result speculates that BMP-2/6 at the low concentration could be sufficient to induce down-signal activation and promote the gene expressions of Runx2 and osterix but may not with BMP-2 or BMP-6. Here, we have demonstrated for the first time that AF cells under HCS stimulation promote osteogenic genes, Runx2, osterix, and OPN by upregulating the BMP-2/6 heterodimer. Furthermore, an increased expression of BMPs associated with excessive mechanical stretch has been shown to occur in disc degeneration [17]. Moreover, our results demonstrated that HCS activated the p38 MAPK and SMAD1/5/8 signal transduction pathway, and these events led to increased Runx2 and osterix expression on AF cells (summarized in Figure 9). Gilbert et al. demonstrated that human AF cells derived from degenerative IVDs stimulated with cyclic tensile strain (CTS) at 10% strain and 1.0-Hz frequency for 20 min resulted in a significant decrease in aggrecan and type I collagen gene expressions [51]. Pratsinis et al. also indicated that AF cells derived from degenerative IVDs stimulated by CTS with a magnitude of 8% (12 h duration at a frequency of 1 Hz) upregulated the expressions of proinflammatory mediators such as COX-2, IL-8, and IL-6 [52]. In addition, stretch-induced inflammatory gene expression may be modulated through the stress-activated protein kinases/Jun NH2-terminal kinases (SAPK/JNKs) and p38 MAPK signaling pathways [52]. According to these findings, HCS may not only induce the inflammatory reaction, but also increase the osteogenic gene expression in AF cells, suggesting that this type of high mechanical stretch at the cellular level could play important roles in DDD progression. Irregular biomechanical loading of the spine and excessive mechanical stretching of tissues and cells are believed to play a major role associated with IVD degeneration.

It has been reported that mechanical stretch can activate MAPK pathways to regulate inflammatory responses in IVD cells [53], but there is no report on the activation of BMP-2/6 heterodimer in AF cells, particularly with HCS. BMPs play a regulatory role in IVD osteogenesis, and there are several reports on the possible linkage between BMP isoforms and the pathogenesis of IVD degeneration [32,54]. Thus, the upregulation of BMP-2/6 heterodimer in human AF cells by HCS could have pathophysiologic significance. Our findings showed that mechanical stretch-induced activation of p38 MAPK and SMAD1/5/8 signaling pathways and consequent expression of Runx2, osterix, and OPN are dependent on upregulation of BMP-2/6 heterodimer and its receptor ALK3, suggesting that cyclic tensile stress may accelerate the progression of IVD degeneration by promoting osteogenic differentiation. Due to insufficient IVD tissue samples collected from patients, we only used commercial human AF cells to investigate the effect under high cyclic stretching. In addition, since the proportion of AF cells in IVD tissues is small, most of them rely on the extracellular matrix (ECM) to maintain the structure of IVD. It is well known that cyclic stretching damages the ECM more severely than AF cells when determining the mechanical effects leading to DDD. However, we may not fully understand the effects of mechanical stretching on IVD tissue. Furthermore, this study did not address the inflammatory response following excessive mechanical stress in AF cells [55], which is also a possible pathological factor associated with IVD osteogenesis. In future experiments, it will be critical to determine whether mechanical stress exerted on AF cells via the ECM can still trigger an osteogenic response.

## 5. Conclusions

Our results provide information regarding the mechanism by which HCS induces osteogenic gene expression in human AF cells. We found that the stimulation of human AF cells with HCS resulted in the activation of the signaling pathways mediated by the BMP-2/6 heterodimer. Inhibition of ALK3 activity may be an effective method to control the osteogenesis of AF cells. HCS also induces activation of the p38 MAPK and SMAD1/5/8 signaling pathways and ultimately enhances the expression of Runx2 and osterix in human AF cells. Furthermore, we have shown that human degenerative IVD tissue has a higher expression of osteogenic genes than LF tissue. These findings provide insight into the mechanisms underlying the interplay of mechanical stretching and cellular signaling in human AF cells, which may be involved in the progression of DDD. Our data provide potentially relevant clues to the mechanisms of IVD degeneration and could be used for future therapeutic interventions for IVD degeneration in patients with DDD.

## Data Availability

The data that support the findings of this study are available until 5 years after publication to researchers who provide a sound proposal and all study sites, and the sponsors agree to share the data. Proposals should be directed toward the corresponding author.

## Supplementary Materials

The following supporting information can be downloaded at: https://www.mdpi.com/article/10.3390/cells11162600/s1, Supplementary original blots were shown below: Figure S1: Original, unedited Runx2, osterix, OPN and β-actin representative blots corresponding to Figure 1; Figure S2: Original, unedited osterix, OPN and β-actin representative blots corresponding to Figure 2; Figure S3: Original, unedited Runx2, osterix and β-actin representative blots corresponding to Figure 3; Figure S4: Original, unedited Runx2, osterix and β-actin representative blots corresponding to Figure 4; Figure S5: Original, unedited Runx2, β-actin, p-p38 and p38 representative blots corresponding to Figure 5; Figure S6: Original, unedited p-SMAD/1/5/8, SMAD1, Runx2 and β-actin representative blots corresponding to Figure 6; Figure S7: Original, unedited Runx2, osterix and β-actin representative blots corresponding to Figure 7; Table S1: The list of antibodies for the Western blot analysis; Table S2; Raw data of real-time PCR from three independent experiments in this study.
