# Supplementary material for: Mechanical Stretch Induced Osteogenesis on Human Annulus Fibrosus Cells through Upregulation of BMP-2/6 Heterodimer and Activation of P38 and SMAD1/5/8 Signaling Pathways
Source: Cells. 2022 Aug 20;11(16):2600. doi: 10.3390/cells11162600 (PMC9406707; doi:10.3390/cells11162600)
Supplement: Supplementary file 1 [file cells-11-02600-s001.zip › cells-1798682-supplementary.pdf]

Figure S1. Original, unedited Runx2, osterix, OPN and β-actin representative blots corresponding to Figure 1.

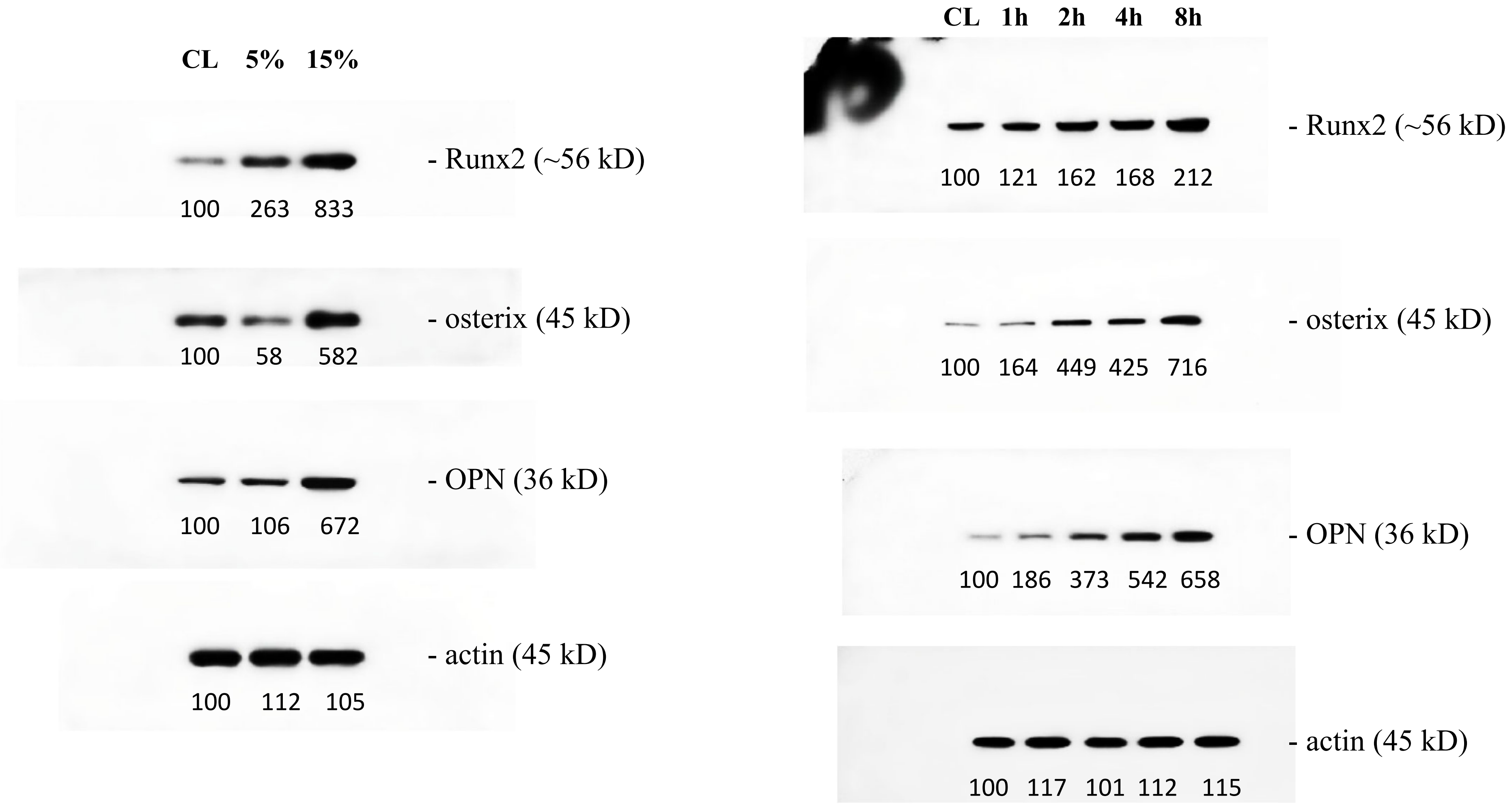

Figure S2. Original, unedited osterix, OPN, and β-actin representative blots corresponding to Figure 2.

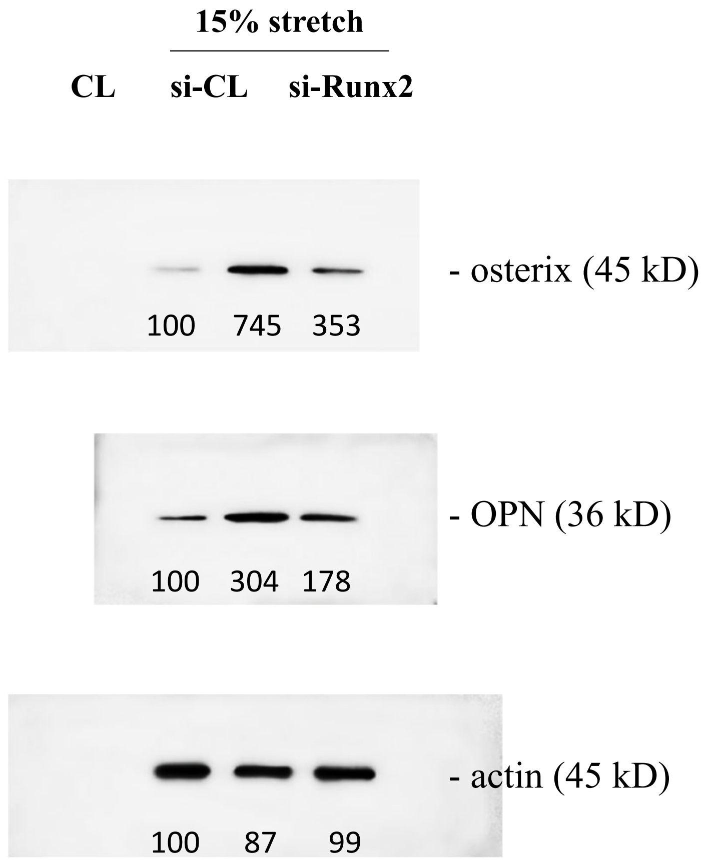

Figure S3. Original, unedited Runx2, osterix, and β-actin representative blots corresponding to Figure 3.

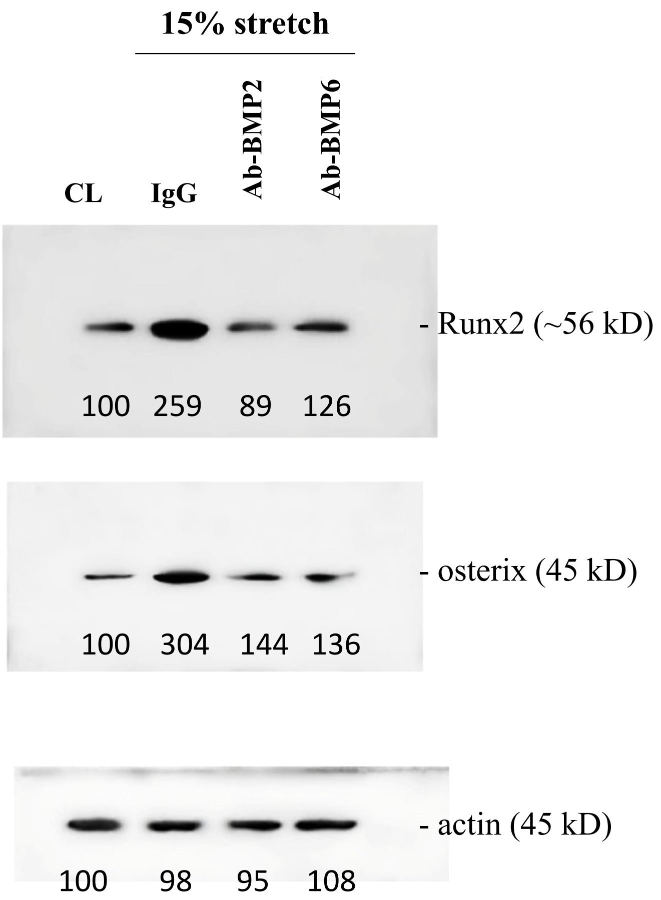

Figure S4. Original, unedited Runx2, osterix, and β-actin representative blots corresponding to Figure 4.

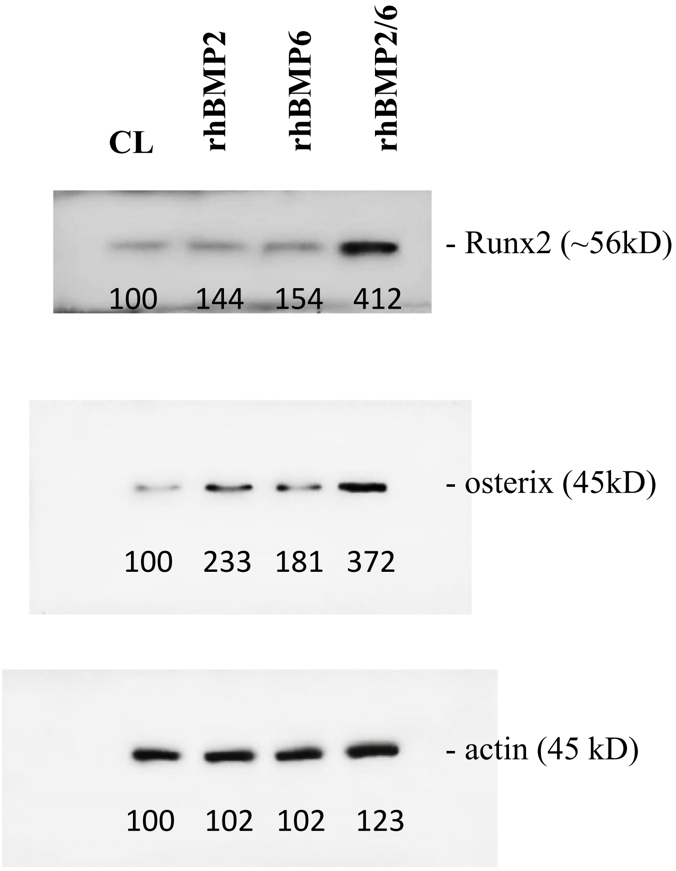

Figure S5. Original, unedited Runx2, β-actin, p-p38, and p38 representative blots corresponding to Figure 5.

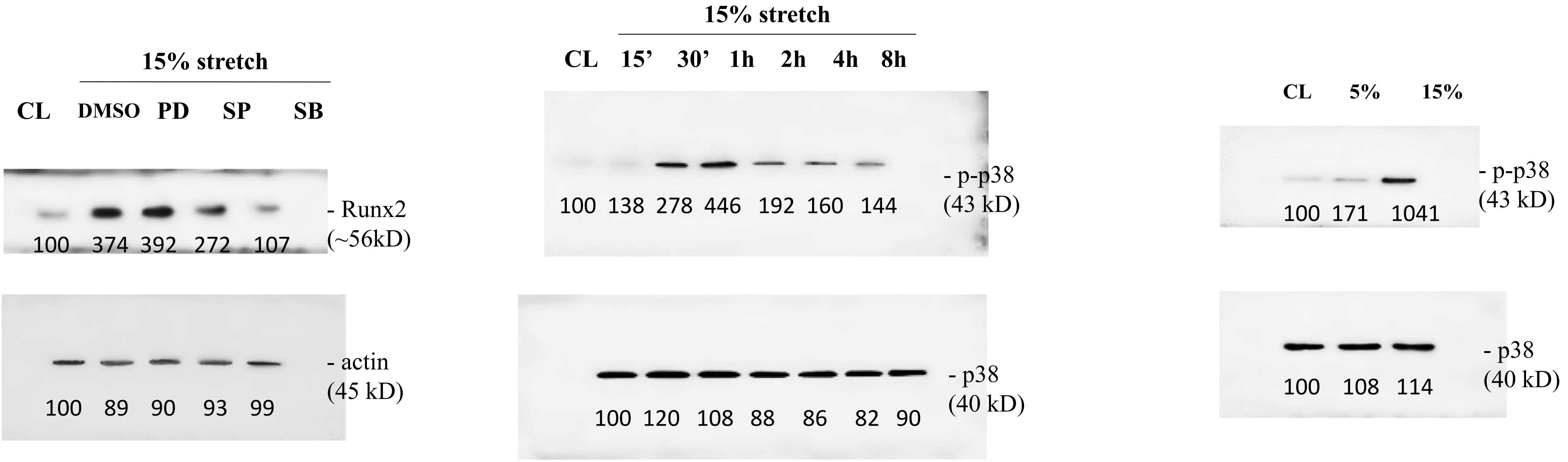

Figure S6. Original, unedited p-SMAD1/5/8, SMAD1, Runx2 and β-actin representative blots corresponding to Figure 6.

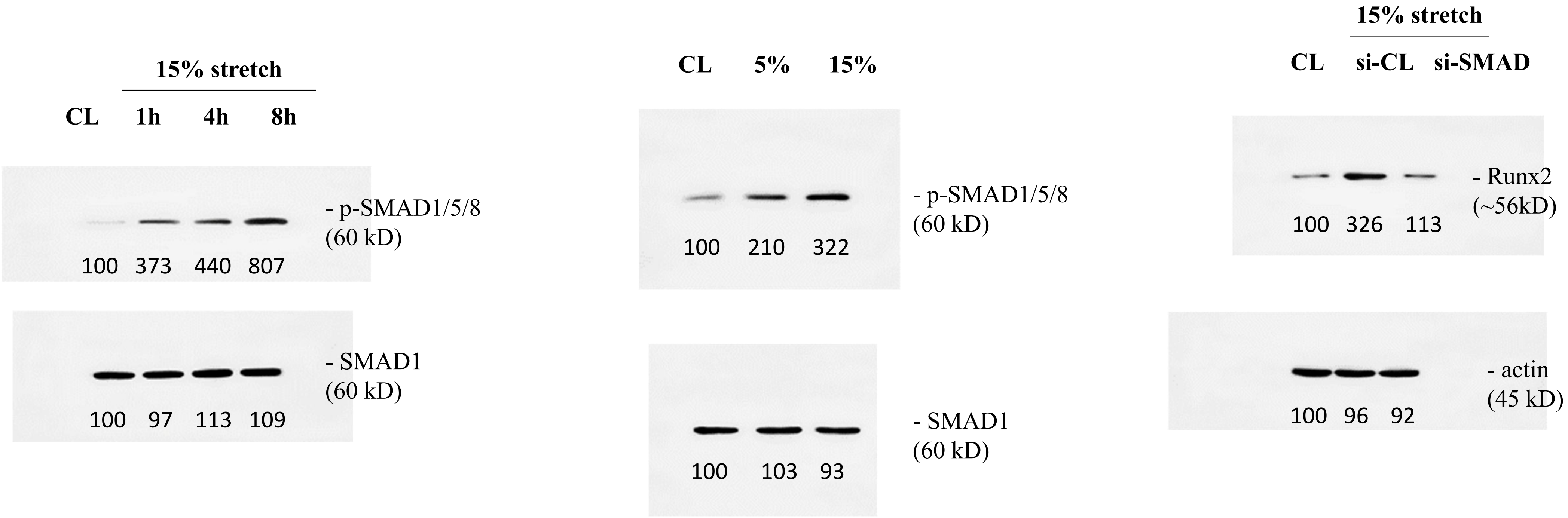

Figure S7. Original, unedited Runx2, osterix, and β-actin representative blots corresponding to Figure 7.

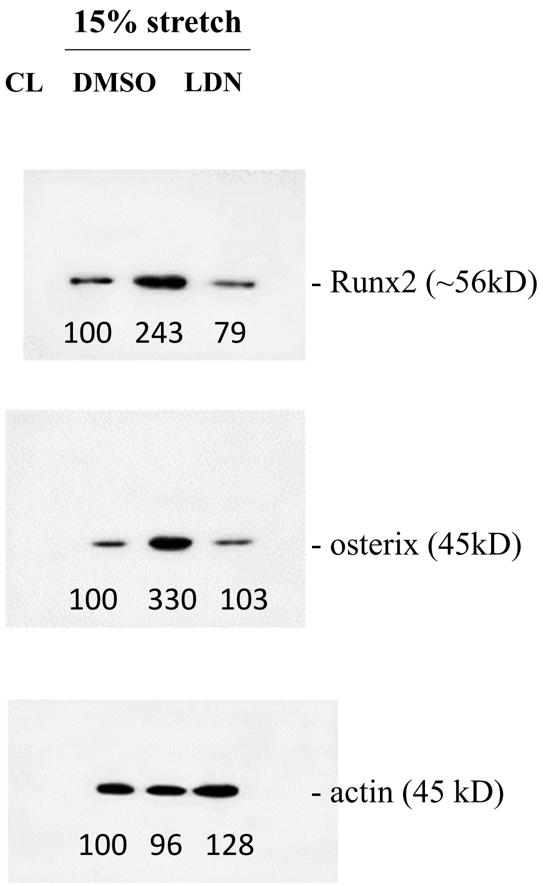

Table S1. List of antibodies for Western blot analysis

**Antibody**	**Dilution**	**Catalog No.**	**Company**
phospho-p38	1:1000	sc-7973	Santa Cruz
p38	1:2500	sc-7972	Santa Cruz
phospho-SMAD1/5/8	1:1000	sc-12353	Santa Cruz
SMAD1/5/8	1:2000	sc-6031-R	Santa Cruz
Runx2	1:2000	#8486	Cell Signaling
osterix	1:1000	sc-393060	Santa Cruz
OPN	1:2500	sc-21742	Santa Cruz
β-actin	1:5000	sc-47778	Santa Cruz

Table S2. Raw data of real time PCR from three independent experiments in this study.

Figure 1A.

	1st batch (fold of CL)	2nd batch (fold of CL)	3rd batch (fold of CL)	Mean	SD
5%-Runx2	1.28	1.62	0.68	1.19	0.47
5%-osterix	0.47	0.88	0.53	0.62	0.22
5%-OPN	1.55	2.18	1.07	1.6	0.55
15%-Runx2	3.11	3.56	2.1	2.92	0.74
15%-osterix	4.11	4.43	2.95	3.83	0.77
15%-OPN	3.26	4.35	2.42	3.34	0.96

Figure 1C.

	1st batch (fold of CL)	2nd batch (fold of CL)	3rd batch (fold of CL)	Mean	SD
1h-Runx2	1.26	1.14	1.76	1.38	0.32
1h-osterix	1.75	1.35	2.32	1.8	0.48
1h-OPN	1.41	2.25	1.31	1.65	0.51
2h-Runx2	1.41	2.1	1.54	1.68	0.36
2h-osterix	1.7	0.84	2.12	1.55	0.65
2h-OPN	3.23	2.12	2.25	2.53	0.60
4h-Runx2	2.34	1.9	2.01	2.08	0.22
4h-osterix	2.32	3.12	2.52	2.65	0.41
4h-OPN	3.62	2.72	3.52	3.28	0.49
8h-Runx2	4.26	3.24	2.82	3.44	0.74

8h-osterix	4.68	3.65	2.98	3.77	0.85
8h-OPN	6.02	6.56	4.68	5.75	0.9

Figure 2A.

	1st batch (fold of CL)	2nd batch (fold of CL)	3rd batch (fold of CL)	Mean	SD
si-CL-osterix	5.05	3.76	3.62	4.14	0.78
si-CL-OPN	4.83	4.02	4.33	4.39	0.40
si-Runx2-osterix	1.84	1.06	1.46	1.45	0.39
si-Runx2-OPN	1.52	0.83	1.68	1.34	0.45

Figure 3A

	1st batch (fold of CL)	2nd batch (fold of CL)	3rd batch (fold of CL)	Mean	SD
BMP2	6.88	6.12	4.65	5.88	1.13
BMP4	0.72	0.86	0.3	0.83	0.1
BMP6	9.82	8.28	4.86	7.65	2.53
BMP7	1.36	1.21	0.62	1.06	0.39

Figure 3B.

	1st batch (fold of CL)	2nd batch (fold of CL)	3rd batch (fold of CL)	Mean	SD
IgG-Runx2	4.48	3.34	3.72	3.85	0.58
IgG-osterix	3.72	5.06	4.31	4.36	0.67
Ab-BMP2-Runx2	1.56	1.16	0.82	1.18	0.37
Ab-BMP2-osterix	1.47	1.38	0.72	1.19	0.41

Ab-BMP6-Runx2	1.28	1.44	1.02	1.24	0.21
Ab-BMP6-osterix	1.46	0.81	1.06	1.11	0.32

Figure 4A.

	1st batch (fold of CL)	2nd batch (fold of CL)	3rd batch (fold of CL)	Mean	SD
rh-BMP2-Runx2	1.52	1.62	1.21	1.45	0.21
rh-BMP2-osterix	0.82	1.16	1.31	1.1	0.25
rh-BMP6-Runx2	1.06	1.65	1.12	1.27	0.32
rh-BMP6-osterix	0.96	1.92	1.13	1.33	0.51
rh-BMP2/6-Runx2	4.42	3.06	3.28	3.58	0.73
rh-BMP2/6-osterix	5.12	4.03	4.46	4.51	0.54

Figure 5A.

	1st batch (fold of CL)	2nd batch (fold of CL)	3rd batch (fold of CL)	Mean	SD
DMSO	3.45	4.32	3.62	3.79	0.46
PD	2.96	4.36	3.24	3.52	0.74
SP	3.68	3.42	2.66	3.25	0.53
SB	1.02	1.62	0.76	1.13	0.44

Figure 6C.

	1st batch (fold of CL)	2nd batch (fold of CL)	3rd batch (fold of CL)	Mean	SD
si-CL	2.88	4.12	3.28	3.42	0.63
si-SMAD	1.34	2.15	1.72	1.74	0.40

Figure 7A.

	1st batch (fold of CL)	2nd batch (fold of CL)	3rd batch (fold of CL)	Mean	SD
si-CL-Runx2	2.68	3.22	3.84	3.24	0.58
si-CL-osterix	4.13	5.52	4.96	4.87	0.69
si-ALK3-Runx2	1.16	1.02	1.51	1.23	0.25
si-ALK3-osterix	1.38	1.82	1.05	1.41	0.38
DMSO-Runx2	4.22	3.28	4.16	3.89	0.52
DMSO-osterix	3.51	4.56	4.94	4.33	0.74
LDN-Runx2	1.04	1.42	1.75	1.4	0.35
LDN-osterix	0.72	1.78	1.41	1.3	0.53

Figure 7C.

	1st batch (fold of CL)	2nd batch (fold of CL)	3rd batch (fold of CL)	Mean	SD
BMP2/6-LDN	5.42	3.94	4.62	4.66	0.72
BMP2/6+LDN	1.67	1.14	1.22	1.34	0.27
